# Efficacy and safety of GLucocorticoid injections into InfrapaTellar faT pad in patients with knee ostEoarthRitiS: protocol for the GLITTERS randomized controlled trial

**DOI:** 10.1186/s13063-022-06993-4

**Published:** 2023-01-03

**Authors:** Yan Zhang, Guangfeng Ruan, Peng Zheng, Sili Huang, Xiaoni Zhou, Xuelian Liu, Wenjie Hu, Huiting Feng, Yangyang Lin, Juanjuan He, Zhenhai Wei, Jiangshan Zhang, Qing Chang, Xiaomei Wei, Tao Fan, Li Jiang, Changhai Ding

**Affiliations:** 1 Clinical Research Centre, Zhujiang Hospital, Southern Medical University, Guangzhou, Guangdong China; 2 Clinical Research Centre, Guangzhou First People’s Hospital, School of Medicine, South China University of Technology, Guangzhou, Guangdong China; 3 Department of Rehabilitation Medicine, Zhujiang Hospital, Southern Medical University, Guangzhou, Guangdong China; 4 Department of Rehabilitation Medicine, Beihai People’s Hospital, Beihai, Guangxi China; 5 Department of Rehabilitation Medicine, The Sixth Affiliated Hospital of Sun Yat-Sen University, Guangzhou, Guangdong China; 6 Department of Rehabilitation, The Third Affiliated Hospital of Sun Yat-Sen University, Guangzhou, Guangdong China

**Keywords:** Osteoarthritis, Glucocorticoid, Infrapatellar fat pad, Randomized controlled trials, Protocol, GLITTERS

## Abstract

**Background:**

Knee osteoarthritis (OA) is a prevalent disabling disorder that involves changes in articular cartilage damage, subchondral bone remodeling, synovitis, and abnormal infrapatellar fat pad (IPFP). Due to the complicated etiology and numerous phenotypes of knee OA, limited improvement is achieved for treatments among knee OA patients with different phenotypes. Inflammatory OA phenotype is a typical knee OA phenotype, and individualized treatment targeting inflammation is a promising way to obtain an optimal therapeutic effect for people with inflammatory knee OA phenotype. Glucocorticoid is a traditional anti-inflammatory drug for knee OA, and intra-articular glucocorticoid injections are recommended clinically. However, emerging evidence has shown that repeated intra-articular glucocorticoid injections in the long term would induce cartilage loss. IPFP and its adjacent synovium are considered as the main source of inflammation in knee OA. This GLITTERS trial aims to investigate if a glucocorticoid injection into the IPFP is effective and safe over 12 weeks among knee OA patients with an inflammatory phenotype.

**Methods:**

GLITTERS is a multicenter, double-blinded, randomized, and placebo-controlled clinical trial among knee OA patients with both Hoffa-synovitis and effusion-synovitis. Sixty participants will be allocated randomly and equally to either the glucocorticoid group or the control group. Each group will receive an injection of glucocorticoid or saline into the IPFP with an intra-articular hyaluronic acid injection as a background treatment at baseline and be followed at 4, 8, and 12 weeks. The primary outcomes will be changes in knee pain on a visual analog scale and effusion-synovitis volume measured on magnetic resonance imaging (MRI). The secondary outcomes will be changes in the total score of Western Ontario and McMaster Universities Osteoarthritis Index score, MRI-detected Hoffa-synovitis score, quality of life, pain medication use, IPFP volume, and the incidence of adverse reactions. Data analyses based on the intention-to-treat principle will include mixed-effects regressions, Wilcoxon rank-sum tests, and chi-square tests (or Fisher’s exact test).

**Discussion:**

GLITTERS may provide high-quality evidence for the efficacy and safety of ultrasound-guided glucocorticoid injections into IPFP among people with inflammatory knee OA in a short term. The results of this trial are expected to provide a reliable reference for a longer-term risk–benefit profile of this treatment in the future.

**Trial registration:**

ClinicalTrials.gov NCT05291650. Registered on 23 March 2022.

## Introduction

Osteoarthritis (OA) is a highly prevalent joint disease characterized by joint pain and structural changes which ultimately lead to loss of joint function [1]. According to the estimation of the World Health Organization, there are about 300 million OA patients worldwide, and the prevalence of OA among people over 50 years old can reach up to 10–20% [2, 3]. Knee OA is the predominant type of OA whose pathological changes include articular cartilage damage, subchondral bone remodeling, synovitis, and abnormal infrapatellar fat pad (IPFP or Hoffa’s fat pad) [4]. Knee OA seriously decreases the quality of life of the patients and leads to a heavy economic burden to patients’ families as well as society [1, 4].

However, there is no curative drug for knee OA. Apart from some patients with end-stage knee OA undergoing total knee arthroplasty, most patients choose conservative treatments to alleviate their symptoms [4]. Due to the complicated etiology and numerous subtypes of knee OA, limited improvement is achieved for similar treatments among patients with different knee OA phenotypes [4]. Therefore, an individualized treatment focusing on a specific phenotype of knee OA is a promising way to obtain an optimal therapeutic effect.

Inflammatory phenotype is one of the most typical phenotypes of knee OA [5–7]. IPFP and synovium as a structural complex are considered as the main source of inflammation in knee OA [8]. IPFP, an adipose tissue, is below the patella and located closely to the synovial layers [9]. Adipocytes and immune cells are abundant in IPFP [9]. Abnormal IPFP can release a variety of inflammatory products which result in changes of the cartilage, synovium, and subchondral bone and eventually accelerate OA progression [10]. Signal changes in IPFP detected by magnetic resonance imaging (MRI) are referred to as Hoffa-synovitis [11]. Effusion-synovitis, a composite of joint fluid and synovial thickening, refers to the MRI-detected signal changes in the synovial cavity. Abundant clinical evidence showed that Hoffa-synovitis and effusion-synovitis were associated with the incidence and progression of knee OA [11–18]. Therefore, anti-inflammatory therapies for those knee OA patients with Hoffa- and effusion-synovitis may have a better clinical effect.

Glucocorticoid is a traditional anti-inflammatory drug for knee OA. It suppresses inflammation via the apoptosis induction of immune cells and the suppression of the proinflammatory mediators’ expression [19]. Clinically, intra-articular glucocorticoid injections are recommended for people with knee OA to reduce their pain [20, 21]. However, emerging evidence has shown that repeated intra-articular injections over 2 years would induce more cartilage loss [22]. Thus, the injection of glucocorticoid into IPFP may not only play a better role in anti-inflammatory but minimize cartilage deterioration among inflammatory knee OA patients. To date, there is no study about the injection of drugs into IPFP.

The characteristics of real-time imaging and tracking in ultrasonography make the injection into inflammatory sites of IPFP possible [23, 24]. Therefore, we hypothesized that the IPFP injection of glucocorticoid can effectively reduce the knee pain and effusion-synovitis volume, compared to the placebo injection among people with symptomatic knee OA who have effusion- and Hoffa-synovitis.

## Objectives

The primary aim is to assess whether the injection of glucocorticoid into the IPFP can effectively reduce the knee pain measured by visual analog scale (VAS) and MRI-measured effusion-synovitis volume, compared to the placebo injection among patients with symptomatic knee OA who have both effusion- and Hoffa-synovitis.

Secondary aims are to evaluate whether the injection of glucocorticoid into the IPFP effectively reduces the total Western Ontario and McMaster Universities Osteoarthritis Index (WOMAC) score, MRI-assessed Hoffa-synovitis score, and pain medication use; improves the quality of life; and has a difference in IPFP volume and adverse reaction, compared to the placebo injection among symptomatic knee OA patients with both effusion- and Hoffa-synovitis.

## Methods and analysis

### Study design

**Fig. 1:**
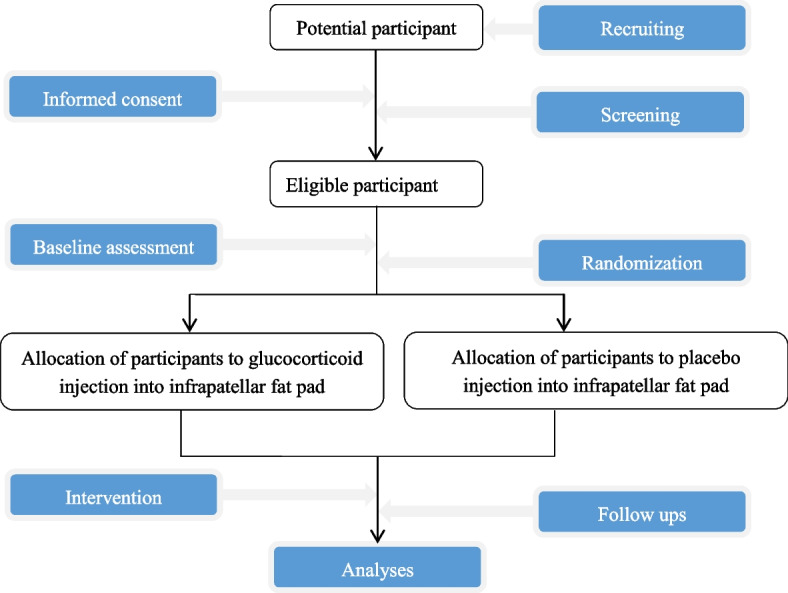
Flowchart of the trial

GLITTERS is a multicenter, randomized, double-blind, placebo-controlled trial over 12 weeks (Fig. 1). The ethics approval in all four centers has been received from the Medical Ethics Committee before the recruitment. The principal study center is Zhujiang Hospital of Southern Medical University (2021-KY-183–02), and the other study centers are the Sixth Affiliated Hospital of Sun Yat-sen University (2022ZSLYEC-179), the Third Affiliated Hospital of Sun Yat-sen University ([2022]02–138-01), and Beihai People’s Hospital (2022–014). This study will use competitive enrollment, and all participants will provide informed written consent prior to data collection. The trial will be reported in accordance with the Consolidated Standards of Reporting Trials guidelines.

### Recruitment and informed consent

The potential participants will be recruited in the following ways: (1) referrals by the participating doctors from outpatient clinics, (2) public education from the surrounding community hospitals, (3) posters/flyers placed in hospitals and surrounding communities, and (4) electronic advertisements on social media platforms. Each participant will be asked to sign two copies of the informed consent to ensure that their participation is voluntary. One copy is preserved by the principal researcher, and the other by the participant.

### Eligibility criteria

The following are the inclusion criteria:

1. Diagnosed with symptomatic knee OA according to American College of Rheumatology criteria [25].
2. Age > 45 years
3. Have knee pain for more than 6 months and the pain assessed by VAS (100 mm) ≥ 40 mm in the last week
4. Ultrasonography showed obvious synovitis (over about 10 ml) with effusion in the knee joint
5. Both MRI-assessed Hoffa-synovitis score (MRI Osteoarthritis Knee Score, MOAKS method) [26, 27] and effusion-synovitis score (MOAKS method) [26] ≥ 1 and their total score ≥ 3
6. Being able to listen, speak, read, and understand Chinese; capable of understanding the study requirements, cooperating with the researchers during the study, and providing written informed consent

The following are the exclusion criteria:

1. Allergy to glucocorticoids
2. Knee joint injection of glucocorticoid or hyaluronic acid within the past 6 months
3. Severe trauma or arthroscopy in the knee within the past 6 months
4. Planned hip or knee surgery (including arthroscopy, arthroplasty, and other open joint surgeries) in the next 6 months
5. Contraindication to having MRI (e.g., implanted pacemaker, artificial metal valve or cornea, aneurysm clipping surgery, arterial dissection, metal foreign bodies in the eyeball, claustrophobia)
6. Presence of other arthritis, such as rheumatoid arthritis and psoriatic arthritis
7. Other conditions that are more painful than knee OA
8. Malignant tumors or other life-threatening diseases
9. Infection, diabetes, coagulopathy, osteonecrosis, or gastric/duodenal ulcer within the past 12 months
10. Current use of oral corticosteroids, non-steroidal anti-inflammatory drugs, or immunosuppressive medication
11. Pregnancy or lactating female
12. Use any investigational drugs or devices in the recent 30 days

Note: When both knees of the participants meet the eligibility criteria, the knee with more severe VAS pain will be selected as the study knee.

### Randomization and blinding

Using block randomization in a block size of four, eligible participants in each center will be assigned to the treatment or placebo group with a 1:1 allocation rate based on computer-generated random numbers. A staff member who is not involved in this trial will pack the drugs with a random number indicated on each of the packages. Allocation concealment will be ensured, and allocation results will be not revealed until the completion of the final data analyses. Double-blind (participants and researchers including outcome assessors and statisticians) design will be applied in this trial. Because of the different appearance of the injection drugs, the physical therapists for the injections are not blinded, and they will not be involved in other processes of this trial. During the period of preparing and injecting the drug, participants will be out of sight of the injection drugs. Unblinding will be conducted after all the data analyses are obtained. Emergency unblinding will be permissible when a serious adverse event happens, and patients who are unblinded will be withdrawn from the trial.

### Intervention

Participants are randomly allocated to either the treatment (glucocorticoid) group or the placebo (saline) group. Each group will receive two injections at baseline: one is the glucocorticoid or saline into IPFP, and the other is intra-articular hyaluronic acid as a background treatment.

The product of the glucocorticoid is betamethasone injectable suspension (Diprosone), and the dosage is 1 ml. To alleviate the discomfort on local tissues, the suspension injected into the IPFP will be pre-mixed with 0.5 ml saline and 0.5 ml lidocaine (concentration: 2%). That is, the total amount of the drug injected into the IPFP for the glucocorticoid group will be 2.0 ml. Accordingly, a total of 2.0 ml drug including 1.5 ml saline and 0.5 ml lidocaine will be injected into the IPFP in the placebo group. The injection of glucocorticoid or placebo into the IPFP will be performed under the guidance of ultrasonography. The procedures are as follows: (1) the participant lies on the bed with a small pillow behind his/her knee to bend the knee joint by 20–30°, (2) the ultrasonic probe is placed under the patella to show the IPFP and its inflammatory sites, (3) insert the needle through the inferior lateral patella, and (4) the drug is injected on two sites at the bottom of IPFP near the synovium where synovial hyperplasia is obvious, with a dosage of 1 ml at each site. All physical therapists will be trained for this injection under the supervision of an experienced therapist.

The intra-articular hyaluronic acid (ARTZ, Seikagaku Corporation, Japan) injection will be added as a background treatment. That is, after completing the process of IPFP injection, the participants in both groups will receive a 2.5-ml hyaluronic acid suspension injection through the suprapatellar bursa into the intra-articular space under the guidance of ultrasonography.

### Outcome measures

An overview of the data collection is listed in Table 1. All questionnaires (VAS, WOMAC, four-dimensional Assessment of Quality of Life (AQoL-4D), and nine-item Patient Health Questionnaire (PHQ-9)) and queries about pain medication use and the use of medication and supplements will be applied at baseline and 4, 8, and 12 weeks of follow-ups. MRI assessments including effusion-synovitis volume, Hoffa-synovitis score, tibiofemoral bone marrow lesion maximum size, tibiofemoral cartilage defects, and IPFP volume will be examined at baseline and 12 weeks of follow-up. Other measures including demographic data, clinical evaluation and history collection, ultrasonographic examination in the study knee, and effusion-synovitis score assessed by MRI will be recorded at baseline/screening only.

**Table 1:** Schedule of the data collection

	**Screening/baseline**	**4 weeks**^a^	**8 weeks**^a^	**12 weeks**
**Primary outcomes**				
Knee pain (VAS)	✔	✔	✔	✔
Effusion-synovitis volume^b^	✔			✔
**Secondary outcomes**				
WOMAC	✔	✔	✔	✔
Hoffa-synovitis score^b^ (MOAKS)	✔			✔
Quality of life (AQoL-4D)	✔	✔	✔	✔
Pain medication use	✔	✔	✔	✔
Infrapatellar fat pad volume^b^	✔			✔
Adverse reaction		✔	✔	✔
**Other measures**				
Demographic data^c^	✔			
Clinical evaluation^d^ and history collection	✔			
Ultrasonographic examination in the study of the knee	✔			
Effusion-synovitis score^b^ (MOAKS)	✔			✔
Tibiofemoral bone marrow lesion maximum size^b^	✔			✔
Tibiofemoral cartilage defect^b^	✔			✔
Use of medication and supplements	✔	✔	✔	✔
Adverse events		✔	✔	✔
Depression (PHQ-9)	✔	✔	✔	✔

*VAS* Visual analog scale, *WOMAC* Western Ontario and McMaster Universities Osteoarthritis Index, *MOAKS* MRI Osteoarthritis Knee Score, *AQoL-4D* four-dimensional Assessment of Quality of Life, *PHQ-9* nine-item Patient Health Questionnaire

^a^The participants will complete the questionnaires (VAS, WOMAC, AQoL-4D, and PHQ-9) of the follow-ups at home and send them back to researchers. As for pain medication use, use of medication and supplements, and adverse events, the researchers will ask the participants via remote interview

^b^In MRI

^c^Including names, gender, birth date, height, weight, and contact details

^d^Diagnosis of symptomatic knee osteoarthritis according to the American College of Rheumatology criteria

### Questionnaires and queries

The VAS (100 mm visual analog scale) [28] will be used to assess knee pain using the standard question: “On this line, how would you rate your knee pain in the last week?” A higher score indicates a higher level of pain severity.

The WOMAC [29] requires patients to rate their pain (five items), stiffness (two items), and functional dysfunction (17 items) in the last week. Each item is a 100-mm visual analog scale. The WOMAC score is calculated by summing the score of each item with every 1 mm representing one point. A higher WOMAC score indicates a more severe OA symptom. When more than five items are not completed, the WOMAC score would be regarded as missing data. Otherwise, the WOMAC score would be calculated by averaging the remaining score and then multiplying by 24.

The AQoL-4D [30] will be used to assess the quality of life in the last week. It comprises four dimensions of independent living, social relationships, psychological well-being, and physical senses. Each dimension has three items with four response categories ranging from 0 to 3. The total AQoL-4D score ranges from 0 to 36, and a higher score indicates a lower quality of life.

The PHQ-9 [31] will be used to assess depression in the last 2 weeks. It comprises nine items with four response categories scored from 0 to 3. The total PHQ-9 score ranges from 0 to 27, and a higher score indicates a more severe depression level.

Queries about medications and supplement use will also be recorded at baseline and each follow-up. Adverse events and reactions will be recorded at each follow-up. Pain medication use would be determined by the researchers from the records of medication use. Adverse events are defined as any untoward event occurring during the trial, regardless of its relation to treatment, and they need to be reported spontaneously when they occur. Details of the adverse event will be recorded and whether it is an adverse reaction will be determined.

### MRI assessments

Sagittal images on intermediate-weighted/proton density-weighted fat suppression sequences will be used in assessing effusion-synovitis volume/score, Hoffa-synovitis score, tibiofemoral bone marrow lesion maximum size, tibiofemoral cartilage defects, and IPFP volume.

Effusion-synovitis volume will be calculated by summing the volume of effusion-synovitis in the suprapatellar pouch, central portion, posterior femoral recess, and submuscular recess using the OsiriX software. Effusion-synovitis score was scored using MOAKS which can be divided into 0–3 grades [26].

Hoffa-synovitis score (MOAKS method) will be assessed according to the discrete area with increased signal intensity in the IPFP on MRI images [26, 27]. It can be divided into 0–3 grades: grade 0 = normal, grade 1 = IPFP with an increase of signal intensity of < 10%, grade 2 = IPFP with an increased signal intensity between 10 and 20%, and grade 3 = IPFP with an increase in signal intensity of > 20%.

Tibiofemoral bone marrow lesions are defined as discrete areas of increased signal in the subchondral bone. Bone marrow lesions’ maximum size will be assessed at the medial tibial, medial femoral, lateral tibial, and lateral femoral compartments. A slice with the greatest area of bone marrow lesions in a specific compartment will be chosen to assess the bone marrow lesions’ maximum size of the corresponding compartment. Bone marrow lesions on adjacent slices will be measured and compared to locate the slice with the maximum lesion size [32]. The tibiofemoral bone marrow lesions’ maximum size will be calculated by summing the maximum lesions’ size of the four compartments.

Cartilage defects will be graded using a modified Outerbridge classification as follows: grade 0 = normal, grade 1 = focal blistering and intra-cartilaginous hyperintensity with a normal contour, grade 2 = irregularities on the surface and loss of thickness of less than 50%, grade 3 = deep ulceration with loss of thickness of more than 50% without exposure of subchondral bone, and grade 4 = full-thickness chondral wear with exposure of subchondral bone [33]. Cartilage defects will be assessed at the medial tibial, medial femoral, lateral tibial, and lateral femoral compartments, and tibiofemoral cartilage defects will be obtained by summing the scores of the four compartments.

IPFP volume will be measured by manually drawing disarticulation contours around the IPFP boundaries using the OsiriX software as reported previously [34].

### Data management

Research Electronic Data Capture (REDCap), a secure web-based application, will be applied to facilitate the data collection throughout the study. It has a function of self-monitoring in data entry which maximizes data quality. After each follow-up, the researcher will timely input the obtained data into REDCap, and a backup REDCap data will be regularly stored at the principal study center. At the completion of the follow-up, the MR images will be assessed. After all the data are obtained, unblinding will be undertaken. In this trial, the two-step unblinding method will be applied. In the first step of unblinding, the participants will be classified into group A and group B for data analyses. After that, the second step of unblinding will assign the two groups to the treatment group or placebo group.

The participants are allowed to withdraw at any time throughout the study. If the participants withdraw from the study before the end of the trial, they are asked to have data collected including an MRI assessment. The reason and date of the withdrawal will be recorded, and the data before the withdrawal will be asked to retain.

### Sample size

We estimated the sample size based on the primary outcomes. The formula *n*_1_ = *n*_2_ = 2 × [(*Z*_*α*_ + *Z*_*β*_) × *σ*/*δ*]^2^ was used to calculate the sample size with an *α* level of 0.05 (two-sided; *Z*_*α*_ = 1.96) and a power of 80% (*Z*_*β*_ = 0.842). It is reported that the minimum clinically important difference (MCID) of knee VAS pain in people with knee OA is 19.9 mm. Assuming that the knee VAS pain reduction in the glucocorticoid injection group compared with the saline injection group is clinically significant, the difference between knee VAS pain changes of the two groups should be at least 19.9 mm (*δ*). As the standard deviation of the change in knee VAS pain from baseline to 3 months follow-up is 24.2 mm (*σ*) in our previous trial [35], the *n*_1_ = *n*_2_ calculated is 24. Considering a 20% loss to follow-up, a sample size of 30 patients in each group is needed.

As an MCID for effusion-synovitis volume has not yet been defined, the detectable difference in effusion-synovitis volume between the treatment and placebo groups was calculated based on the given sample size (*n*_1_ = *n*_2_ = 30) and *σ*. According to the standard deviation of the change in effusion-synovitis volume from baseline to 12 weeks of follow-up being 7.69 ml (*σ*) in the same previous trial [36], the detectable difference of effusion-synovitis volume calculated is 6.22 ml.

### Statistical plan

Baseline characteristics will be displayed in descriptive statistics according to the data type. Continuous variables with normal distribution will be displayed by means and standard deviation. Continuous variables with non-normal distribution will be displayed by median and interquartile range. Category variables will be displayed by proportion.

The intention-to-treat analysis will be the primary analysis method. The per-protocol analysis will be the second analysis method where the per-protocol population is defined as participants who completed four follow-ups without major protocol deviations. There is no interim analysis in this research. Missing data due to dropout and non-responses will be addressed using multiple imputations with chain equations. For each treatment group, imputations will be performed separately based on the baseline characteristics and the non-missing values at other time points of the missing variables with the assumption of data missing at random. Imputations will be performed separately for each treatment group and each outcome using baseline variables of age, sex, BMI, and study site and non-missing values of the outcomes at baseline and each follow-up with the assumption of data missing at random.

Mixed-effects regression models will be used to calculate treatment group differences with continuous measures. In the models, fixed effects will be follow-up time, baseline covariates (including age, sex, BMI, baseline value of the corresponding outcome), treatment, and interactions between follow-up time and baseline covariates and treatment. Study site and individual participant identification will be treated as the random intercepts and follow-up time as the random slop in the models. The overall treatment group differences will be calculated by the linear combination of the estimated coefficients. Wilcoxon rank-sum test will be used to analyze the difference in pain medication use between the treatment and placebo groups. Pain medication use will be classified as commenced/increased, unchanged, or discontinued/decreased. The chi-square test or Fisher’s exact test will be used to compare the incidence of adverse reactions between the two treatment groups, and the number of adverse reactions will be reported as the number of participants reporting at least one adverse reaction. All analyses will be performed using Stata, version 15 (StataCorp). A 2-sided *P* value of 0.050 will be treated as statistically significant.

## Discussion

The GLITTERS trial is to investigate the efficacy and safety of glucocorticoid injections into IPFP in patients with inflammatory knee OA over 12 weeks. Drug injections are usually administrated in the joint cavity of knee OA patients, yet long-term intra-articular glucocorticoid injections were reported to induce cartilage loss. Given that IPFP near the synovium is the site of inflammation, the glucocorticoid injection into IPFP may maximize the efficacy and minimize the cartilage damage of the glucocorticoid. As this is the first study about the injection of glucocorticoid into IPFP, potential adverse reactions such as fat pad atrophy are noteworthy and therefore IPFP volume is set as one of the secondary outcomes. In other respects, shrinking the size of IPFP may not be unfavorable because abundant evidence including our previous studies has shown that abnormal IPFP may play a damaging role in knee OA [27, 37]. A systematic review on total knee replacement demonstrated that there was no difference in pain and function between IPFP preservation and resection [38]. On balance, a single injection into the IPFP in GLITTERS is not a serious concern.

To improve patient recruitment and obtain ethical approval, we added intra-articular hyaluronic acid injection as a background treatment in both groups. Hyaluronic acid is a major component of synovial fluid and serves as a lubricant within the knee joint. In the process of OA, the synovial fluid would decrease which is associated with joint pain and functional impairment [39]. Exogenous hyaluronic acid injections have therefore been employed clinically to attenuate the macerated activities of OA patients’ depolymerized endogenous hyaluronic acid [40]. Though some discrepancies between studies exist, the majority are overwhelmingly positive for the intra-articular hyaluronic acid injection among knee OA patients [39]. A recent systematic review concluded that intra-articular hyaluronic acid injection had moderate symptomatic benefits without major safety concerns [41]. Of note, because the participants in GLITTERS have a certain amount of effusion in the joint cavity, the process of injection should be sufficiently slow and attentive to guarantee a stable pressure. With regard to participants with an abundant amount of effusion where the excessive effusion is squeezed into the syringe, we will discard this effusion and then inject hyaluronic acid suspension.

In summary, GLITTERS is the first study assessing the efficacy and safety of ultrasound-guided glucocorticoid injections into IPFP among people with inflammatory knee OA in a short term. GLITTERS’s scientific and rigorous methodological design is expected to provide a reliable reference for a longer-term risk–benefit profile of this treatment in the future.

## Trial status

At the time of submitting this manuscript, the study is ongoing (11–02-2022, version 2) and had been actively recruiting participants. The inclusion of the first participant was on 25 April 2022. An expected date that recruitment will be completed is the end of April 2023.

## Data Availability

Data will be available from the corresponding author upon reasonable request.

## Abbreviations

OA: Osteoarthritis
IPFP: Infrapatellar fat pad
MRI: Magnetic resonance imaging
VAS: Visual analog scale
WOMAC: Western Ontario and McMaster Universities Osteoarthritis Index
MOAKS: MRI Osteoarthritis Knee Score
AQoL-4D: Four-dimensional Assessment of Quality of Life
PHQ-9: Nine-item Patient Health Questionnaire
REDCap: Research Electronic Data Capture
MCID: Minimum clinically important difference
